# The neuroprotective effects of AMN082 on neuronal apoptosis in rats after traumatic brain injury

**DOI:** 10.1186/s12868-021-00649-w

**Published:** 2021-06-25

**Authors:** Chung-Che Lu, Tee-Tau Eric Nyam, Jinn-Rung Kuo, Yao-Lin Lee, Chung-Ching Chio, Che-Chuan Wang

**Affiliations:** 1 Department of Neurosurgery, Chi-Mei Medical Center, 901 Chung Hwa Road, Yung Kang City, Tainan, Taiwan; 2 Department of Medical Research, Chi-Mei Medical Center, Tainan, Taiwan; 3 Center for General Education, Southern Taiwan University of Science and Technology, Tainan, Taiwan

**Keywords:** AMN082, Traumatic brain injury, Nitrosative stress, Apoptosis, NMDA receptor

## Abstract

**Background:**

The aim of this study was to investigate whether AMN082 exerts its neuroprotective effect by attenuating glutamate receptor-associated neuronal apoptosis and improving functional outcomes after traumatic brain injury (TBI).

**Methods:**

Anesthetized male Sprague–Dawley rats were divided into the sham-operated, TBI + vehicle, and TBI + AMN082 groups. AMN082 (10 mg/kg) was intraperitoneally injected 0, 24, or 48 h after TBI. In the 120 min after TBI, heart rate, mean arterial pressure, intracranial pressure (ICP), and cerebral perfusion pressure (CPP) were continuously measured. Motor function, the infarct volume, neuronal nitrosative stress-associated apoptosis, and *N*-methyl-d-aspartate receptor 2A (NR2A) and NR2B expression in the pericontusional cortex were measured on the 3rd day after TBI.

**Results:**

The results showed that the AMN082-treated group had a lower ICP and higher CPP after TBI. TBI-induced motor deficits, the increase in infarct volume, neuronal apoptosis, and 3-nitrotyrosine and inducible nitric oxide synthase expression in the pericontusional cortex were significantly improved by AMN082 therapy. Simultaneously, AMN082 increased NR2A and NR2B expression in neuronal cells.

**Conclusions:**

We concluded that intraperitoneal injection of AMN082 for 3 days may ameliorate TBI by attenuating glutamate receptor-associated nitrosative stress and neuronal apoptosis in the pericontusional cortex. We suggest that AMN082 administration in the acute stage may be a promising strategy for TBI.

## Introduction

Excessive glutamate excitotoxicity is well known to be a leading cause of neurological complications after traumatic brain injury (TBI) [1]. Metabotropic receptors (mGluRs) [2] and ionotropic receptors are two major glutamate receptors in the central nervous system (CNS) [3, 4]. According to molecular cloning data, there are eight different metabotropic receptor subtypes, i.e., mGluR1 to mGluR8, with different molecular structures and pharmacological properties [2]. Among ionotropic receptors, *N*-methyl-d-aspartate receptor 2A (NR2A) and 2B (NR2B) are two major NMDA receptors [3, 4]. Triggering of astroglial and neuronal glutamate excitotoxicity generates inducible nitric oxide synthase (iNOS) and induces reactive nitrosative stress (RNS), eventually contributing to cell apoptosis [5, 6]. Therefore, inhibition of glutamate-related RNS processes may be a therapeutic strategy for TBI.

Although there is little data in the literature, some authors have reported changes in the expression of mGluRs in TBI. Gong et al. showed that at 7 days after TBI, mGluR2/3 and mGluR5 expression was reduced in the ipsilateral hippocampus and cortex [7]. Fei et al. demonstrated a significant rise in the expression of mGluR1 and mGluR5 (peaking at 24 h) and mGluR4, mGluR7 and mGluR8 (peaking at 6 h) and a decrease in the expression of mGluR2 and mGluR3 (peaking at 24 h) after diffuse brain injury [8, 9]. Therefore, modification of the expression of mGluR may provide neuroprotection after brain injury.

AMN082 (*N*,*N*′-dibenzhydrylethane-1,2-diamine dihydrochloride) is a blood–brain barrier-permeable and highly selective mGLu7 receptor allosteric agonist with clear pharmacokinetic activities [10]. Previously, the neuroprotective effects of AMN082 against the neurotoxic and proapoptotic effects of sevoflurane were demonstrated in both in vitro and in vivo models [11]. Moreover, AMN082 has been found to exert neuroprotective effects against oxygen–glucose deprivation- and kainate-evoked neuronal cell damage in in vitro models [12]. The neuroprotective and glioprotective effects of AMN082 have also been observed in models of apoptotic (induced by staurosporine and doxorubicin) and apoptotic-necrotic [induced MPP (+)] damage [13–16].

Furthermore, AMN082 has been shown to affect the CNS to exert anti-anxiety, antidepressive effects [17–19] and antiparkinsonian-like effects [20], and it is used in the treatment of cocaine or opioid addiction [21]. Its mechanisms include regulating glutamate release at the synapse [22], binding to the serotonin transporter [10], activating prosurvival MAPK/ERK 1/2 and PI3-K/Akt pathways [15], and stimulating mammalian target of rapamycin (mTOR) activation [18].

However, to date, AMN082 has not been applied for the treatment of TBI. The effects of AMN082 on neurons and glia as well as TBI-induced nitrosative stress-associated neuronal apoptosis have not been well investigated.

In the current study, we hypothesized that AMN082 has therapeutic effects against TBI because it is a selective mGlu7 receptor that affects glutamate release. We aimed to elucidate the beneficial effects of AMN082 against TBI-induced nitrosative stress-related apoptosis. We hope our results provide new evidence that AMN082 may play a role in clinical treatment in the field of neurotrauma in the future.

## Methods

### Experimental design

**Fig. 1:**
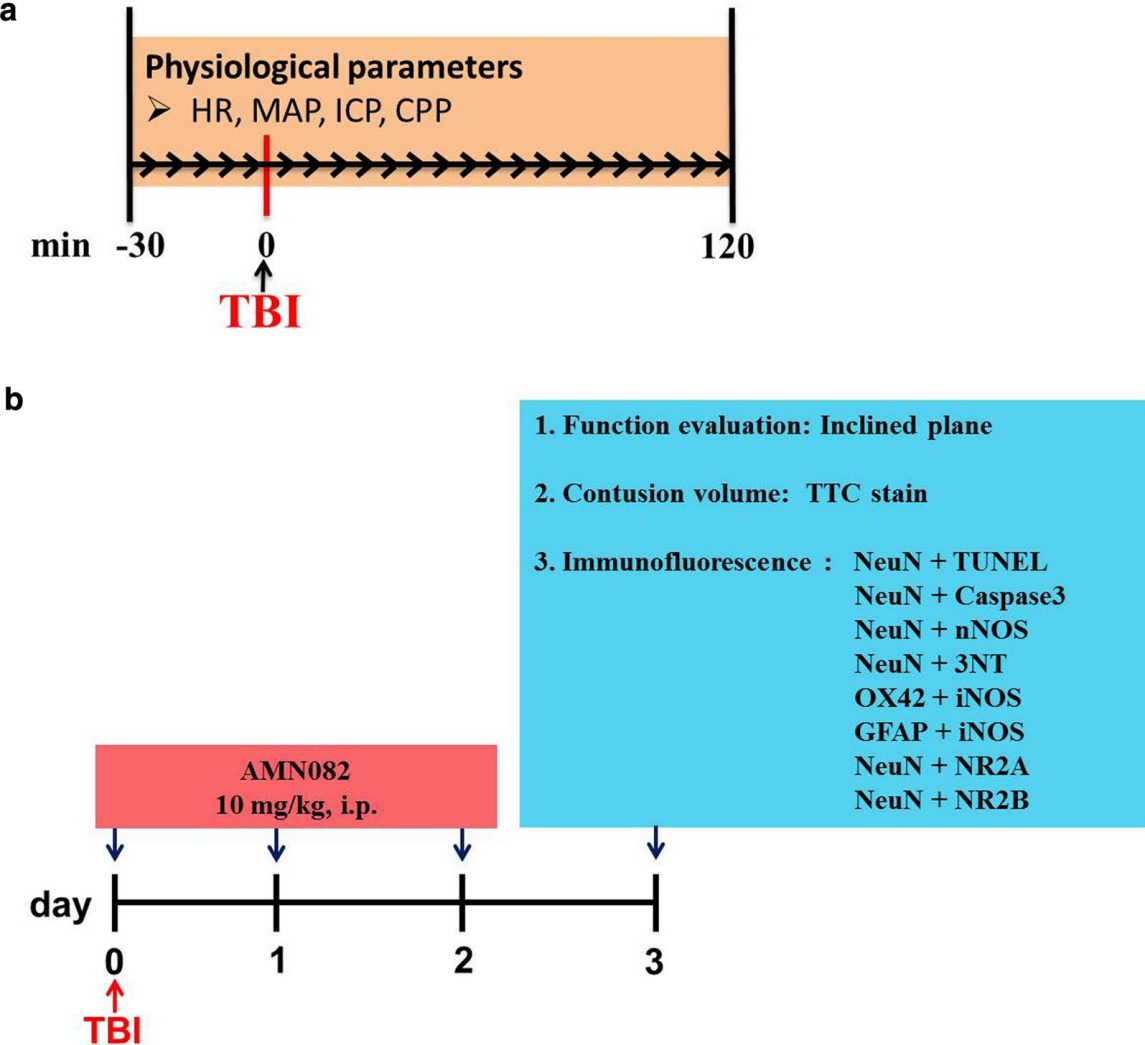
Summary of the experimental protocol

The overall experimental protocol is shown in Fig. 1. First, we continuously measured physiological changes, including heart rate (HR), mean arterial pressure (MAP), intracranial pressure (ICP) and cerebral perfusion pressure (CPP), for 120 min after TBI. Then, we tested whether the changes induced by TBI were associated with nitrosative stress-related neuronal apoptosis and cerebral infarction on the ipsilateral side of the cortex using immunofluorescence and TTC staining. Motor function was assessed by the inclined plane test on the 3rd day after TBI. At the same time point, the effects of AMN082 on TBI-related parameters were also evaluated. All methods were carried out in accordance with relevant guidelines and regulations.

### Animals

Ten-week-old adult male Sprague–Dawley rats were obtained from a commercial source (BioLASCO Taiwan Co., Ltd.) and used in the experiments. The animals were housed under a 12/12-h light/dark cycle and provided free access to food and water. The Chi Mei Medical Center’s Animal Care and Use Committee approved all of the experimental procedures, which conformed to the NIH guidelines, including minimization of discomfort to the animals during surgery and during the recovery period (approval no. 108080602). At the end of the experiments, the rats were euthanized by an overdose of urethane (intraperitoneal injections of 2 ml of 500 mg/ml urethane solution) until deep unconscious condition determined by the absence of visible breathing. The study was carried out in compliance with the ARRIVE guidelines.

### Traumatic brain injury

The animals were anesthetized with a mixture of ketamine (44 mg/kg, i.m.; Nankuang Pharmaceutical, Taiwan), atropine (0.063 mg/kg, i.m.; Sintong Chemical Ind. Co., Taiwan), and xylazine (6.77 mg/kg, i.m.; Bayer, Germany). On a stereotaxic frame (Kopf 1406; Grass Instrument, Quincy, MA), 2-mm-radius craniectomy was performed 3 mm from the sagittal suture and 4 mm from bregma above the right parietal cortex. After implantation of the injury cannula, a fluid percussion device (VCU Biomedical Engineering, Richmond, VA, USA) was connected to the craniectomy site via Luer-lock fitting, and the brain was subjected to percussion injury at 2.2 atm for 25 ms as previously described by Mclntosh et al. [23]. The selection criteria for successful TBI in the rats were transient hypertensive response, apnea, and seizure observed immediately following fluid percussion injury.

### Surgery and physiological parameter monitoring

The right femoral artery of each rat was cannulated with polyethylene tubing (PE50) for blood pressure monitoring. The mean arterial pressure (MAP) and heart rate (HR) were monitored continuously after TBI with a pressure transducer. An intracranial pressure (ICP) microsensor (Codman and Shurtlef, Inc., Rayman, MA, USA) was placed in the parenchyma of the left frontal lobe of each rat. The ICP was monitored continuously, and ICP and cerebral perfusion pressure (CPP) values were recorded at 5-min intervals for 120 min after TBI. The CPP is the MAP-ICP [24]. Colon temperatures were measured with an analog electronic thermometer (model 43 TE; YSI, Inc, Yellow Springs, OH, USA) and temperature probe (series 400; YSI, Inc).

### Treatment intervention

Using a random number table, the rats were randomly assigned numbers and divided into three groups: the sham operated group (n = 6); the vehicle (dimethylsulfoxide (DMSO), 4%, 1 ml/kg, intraperitoneal; K42088831, Merck, Darmstadt, Germany)-treated TBI control group; and AMN082 (10 mg/kg, dissolved in DMSO, intraperitoneal; U.S. Pharmacopeia)-treated TBI group (n = 6). The vehicle-treated TBI group received an equal volume of vehicle via intraperitoneal injection. Vehicle or AMN082 was administered for three consecutive days after TBI. The first injection occurred immediately after TBI, the second injection was administered 24 h later, and the third injection was administered 48 h later. All tests were performed by investigators and assessors blinded to the experimental groups, which were revealed only at the end of the analyses. Table 1 provides detailed information about animal grouping and principal component analysis.

**Table 1:** Detailed information about animal grouping and principal component analysis

Group	Parameter		
	Physiological parameters	Inclined plane, TTC stain	IF stain
Sham	72 h after craniectomy n = 6	72 h after craniectomy n = 6	72 h after craniectomy n = 6
TBI + vehicle	72 h after TBI n = 6	72 h after TBI n = 6	72 h TBI n = 6
TBI + AMN082	72 h after TBI n = 6	72 h after TBI n = 6	72 h after TBI n = 6

### Motor function test

An inclined plane was used to measure limb strength in the motor function test [25]. This test assesses the motor function of rats after neural injury by evaluating a rat’s ability to prevent itself from falling and the endurance strength of the upper and lower limbs on an inclined plane. The animals were initially placed on a 20 × 20-cm inclined plane with a ribbed surface and a 30° angle. To determine the maximal angle at which an animal could remain on the inclined plane without falling, the angle was increased in 1° increments. Motor deficits were assessed by determining the mean maximal angles of the left upper and lower limbs on the 3rd day after TBI.

### Cerebral infarction assay

Triphenyltetrazolium chloride (TTC) staining was performed as described elsewhere [26]. Briefly, brain tissues were removed, immersed in cold saline for 5 min, and sliced into 1-mm-thick sections. The brain slices were incubated in 2% TTC dissolved in phosphate-buffered saline for 30 min at 37 °C and then transferred to 10% formaldehyde solution for fixation. The volume of infarction, as revealed by negative TTC staining, which indicated dehydrogenase-deficient tissue, was measured in each slice and summed using computerized planimetry (PC-based Image Tools software). The volume of infarction was calculated as 1 mm (the thickness of the slice) × (the sum of the infarct area in all brain slices [mm^2^]) [27].

### Immunofluorescence assay

Immunofluorescence was performed on the 3rd day after TBI [28]. Adjacent 6-μm sections corresponding to the region 0.20–0.70 mm anterior to bregma were incubated in 2 mol/L HCl for 30 min, rinsed in 0.1 mol/L boric acid (pH 8.5) for 3 min at room temperature, and then incubated with primary antibodies in PBS containing 0.5% normal bovine serum at 4 °C overnight. After being washed in PBS, the sections were incubated with secondary antibodies for 1 h at room temperature. The number of positive cells in the pericontusional cortex was calculated using computerized planimetry (400 × magnification, Image-Pro Plus Media Cybernetics, Inc. Washington Street, Rockville, USA), and the number of positive cells per mm^2^ of the region of interest is presented. Details about the antibodies used are provided in Table 2.

**Table 2:** The detailed information of antibodies used in current study

Antibody	Source	Catalog number	Working dilution
Mouse Anti-NeuN	Abcam (Cambridge, MA)	ab104224	1:600
Mouse Anti-OX42	Abcam (Cambridge, MA)	ab1211	1:500
Mouse Anti-GFAP	cell signaling technology (Beverly, MA)	3670	1:800
Rabbit anti-Caspase3	cell signaling technology (Beverly, MA)	9661	1:500
Rabbit anti-nNOS	Invitrogen (Eugene, Oregon)	PA3-032A	1:200
Rabbit anti-iNOS	Abcam (Cambridge, MA)	ab15323	1:200
Rabbit anti-NMDAR2A	Chemicon international (Billerica, MA)	AB1555	1:600
Rabbit anti-NMDAR2B	Abcam (Cambridge, MA)	ab65783	1:500
Goat anti-Mouse AF594	Abcam (Cambridge, MA)	ab150116	1:800
Goat anti-rabbit AF488	Abcam (Cambridge, MA)	ab150077	1:400

### Statistical analysis

According to analysis of variance (ANOVA) with a type I error of 0.05 and power calculation for the smallest effect size at 0.2, at least 6 rats per group were needed. This proposed sample size was sufficient to detect a significant difference by ANOVA at the 0.05 level with a power of 80%. All of the data in this study were analyzed using SigmaPlot, version 10.0, for Windows (Systat Software, San Jose, CA, USA). The results of the experiments are expressed as the means ± standard deviations of the means. For each time point, the mean difference between the three groups was analyzed using ANOVA followed by Scheffe’s post hoc test was used to analyze the significance of the differences between the three groups. p-values < 0.05 were considered statistically significant.

## Results

### Basic data for the experimental rats

A total of 54 10-week-old male rats weighing 413 ± 8.7 g were used in the experiments. The fluid percussion force was 2.20 ± 0.01 atm. The colon temperature was maintained at ~ 36–37 °C with a lamp during the procedure and for up to 120 min after injury. No animals died over the course of the experiments. Table 2 provides detailed information about animal grouping and principal component analysis.

### Trend of a lower ICP and higher CPP in the AMN082-treated group compared with the TBI + vehicle group in the initial 120 min after TBI

The ICP was higher in the TBI group than in the sham-operated control group from 0 to 120 min after the start of FPI. In contrast, the CPP was significantly lower in the TBI group than in the sham-operated control group (*p < 0.05; n = 6 in the sham group and n = 6 in the TBI group; Table 3).

**Table 3:** Effects of AMN082 on TBI-induced (a) heart rate (HR), (b), mean arterial pressure (MAP), (c) intracranial pressure (ICP), and (d) cerebral perfusion pressure (CPP) during the 120 min after traumatic brain injury

Time (min)	− 5	0	5	10	15	20	25	30	35	40	45	50	55	60	65	70	75	80	85	90	95	100	105	110	115	120
(a) Heart rate (bpm)																										
Sham	267 ± 12.4	258 ± 11.2	261 ± 9.6	256 ± 9.8	259 ± 8.8	261 ± 11.0	262 ± 10.5	264 ± 10.2	259 ± 9.2	257 ± 8.8	259 ± 11.0	257 ± 8.4	252 ± 9.3	253 ± 8.5	252 ± 9.5	258 ± 12.9	256 ± 9.4	253 ± 9.0	254 ± 9.2	253 ± 9.2	258 ± 10.5	265 ± 14.4	266 ± 12.1	265 ± 12.6	264 ± 15.4	263 ± 13.7
TBI + vehicle	285 ± 24.8	288 ± 22.6	231 ± 8.5*	261 ± 23.1	271 ± 19.8	256 ± 9.9	245 ± 9.0	236 ± 7.0*	234 ± 9.1	236 ± 10.1	239 ± 11.9	237 ± 11.8	239 ± 12.6	240 ± 11.1	241 ± 11.6	241 ± 12.4	239 ± 11.0	245 ± 11.7	249 ± 12.6	250 ± 10.6	258 ± 9.1	256 ± 11.3	255 ± 11.3	257 ± 10.9	260 ± 12.0	262 ± 12.7
TBI + AMN082	288 ± 18.1	262 ± 18.6	234 ± 9.8	252 ± 14.9	255 ± 16.7	250 ± 11.8	235 ± 7.8	233 ± 6.7#	229 ± 6.5#	231 ± 6.5#	224 ± 8.2#	229 ± 6.3	230 ± 7.1	234 ± 7.2	231 ± 9.0	234 ± 5.5	234 ± 7.2	239 ± 5.1	235 ± 5.7	234 ± 6.4	232 ± 5.2#,$	237 ± 6.1	241 ± 5.4	241 ± 5.5	242 ± 4.5	243 ± 4.2
(b) Mean arterial pressure (mmHg)																										
Sham	98 ± 3.3	96 ± 3.5	96 ± 3.3	97 ± 3.2	95 ± 2.6	98 ± 3.7	96 ± 3.2	95 ± 2.3	93 ± 2.7	92 ± 2.8	92 ± 2.5	90 ± 2.6	91 ± 3.2	93 ± 2.6	93 ± 2.5	93 ± 2.2	89 ± 2.6	91 ± 2.5	89 ± 2.8	89 ± 2.5	90 ± 2.2	90 ± 2.4	90.0 ± 2.6	89.8 ± 1.5	90.9 ± 2.6	90.8 ± 2.2
TBI + vehicle	105 ± 3.5	139 ± 4.8***	117 ± 5.9**	104 ± 4.7	96 ± 4.2	94 ± 2.8	91 ± 2.8	89 ± 1.9	89 ± 2.0	88 ± 2.1	88 ± 1.3	89 ± 1.5	87 ± 1.3	88 ± 1.7	87 ± 2.2	86 ± 2.6	85 ± 1.5	87 ± 1.9	89 ± 3.2	91 ± 3.6	89 ± 2.4	90 ± 2.9	90 ± 3.3	90 ± 2.9	91 ± 3.5	92 ± 2.7
TBI + AMN082	102 ± 5.1	142 ± 9.5###	124 ± 5.0###	110 ± 4.7##	102 ± 4.4	97 ± 4.2	94 ± 4.4	93 ± 4.2	94 ± 4.2	92 ± 3.7	92 ± 4.0	92 ± 3.4	90 ± 3.5	92 ± 3.8	91 ± 3.3	90 ± 3.4	90 ± 3.6	90 ± 2.9	90 ± 2.9	89 ± 3.3	89 ± 3.4	91 ± 3.2	92 ± 2.5	92 ± 2.7	94 ± 2.0	93 ± 2.3
(c) Intracranial pressure (mmHg)																										
Sham	8 ± 0.7	8 ± 0.7	8 ± 0.8	8 ± 0.7	8 ± 0.7	8 ± 0.7	8 ± 0.8	8 ± 0.8	8 ± 0.8	8 ± 0.9	8 ± 0.9	8 ± 0.9	8 ± 0.9	8 ± 0.9	8 ± 0.9	8 ± 0.9	8 ± 0.9	8 ± 0.9	8 ± 0.9	8 ± 0.9	8 ± 0.9	8 ± 0.9	8 ± 0.9	8 ± 0.9	8 ± 0.9	8 ± 0.9
TBI + vehicle	9 ± 1.4	23 ± 4.3**	13 ± 2.4	11 ± 2.0	11 ± 2.0	11 ± 1.9	11 ± 1.8	11 ± 1.8	11 ± 1.7	11 ± 1.7	11 ± 1.7	11 ± 1.7	12 ± 1.7	12 ± 1.7	12 ± 1.7	12 ± 1.7	12 ± 1.7	12 ± 1.7	12 ± 1.6	12 ± 1.6*	12 ± 1.6*	12 ± 1.6*	12 ± 1.6*	12 ± 1.6*	12 ± 1.6	12 ± 1.6
TBI + AMN082	9 ± 1.7	20 ± 3.5##	14 ± 3.0	12 ± 2.4	12 ± 2.1	11 ± 1.9	10 ± 1.9	10 ± 1.9	10 ± 1.7	10 ± 1.7	10 ± 1.7	10 ± 1.7	10 ± 1.7	10 ± 1.7	10 ± 1.7	10 ± 1.7	10 ± 1.7	10 ± 1.7	10 ± 1.7	10 ± 1.7	10 ± 1.7	10 ± 1.7	10 ± 1.7	10 ± 1.7	10 ± 1.7	10 ± 1.7
(d) Cerebral perfusion pressure (mmHg)																										
Sham	90 ± 3.3	89 ± 3.6	88 ± 3.3	89 ± 3.2	88 ± 2.7	91 ± 3.6	88 ± 3.4	87 ± 2.5	85 ± 3.0	85 ± 3.2	84 ± 2.5	82 ± 2.9	83 ± 3.6	85 ± 3.2	86 ± 2.8	85 ± 2.8	82 ± 2.9	83 ± 2.5	81 ± 3.0	82 ± 2.7	82 ± 2.4	82 ± 2.5	83 ± 2.8	82 ± 1.7	83 ± 2.9	83 ± 2.4
TBI + vehicle	95 ± 2.9	116 ± 5.9**	105 ± 6.3*	92 ± 4.7	85 ± 3.7	82 ± 3.1	80 ± 2.7	78 ± 2.1*	78 ± 2.5	77 ± 2.7	77 ± 2.1	78 ± 1.9	75 ± 1.9	76 ± 2.0*	76 ± 2.5*	74 ± 3.2*	74 ± 2.0*	75 ± 1.7*	77 ± 3.5	80 ± 3.8	77 ± 2.5	79 ± 3.2	79 ± 3.5	79 ± 3.3	80 ± 3.9	81 ± 2.7
TBI + AMN082	92 ± 4.6	122 ± 7.6##	110 ± 4.7##	98 ± 5.2	90 ± 4.4	86 ± 4.7	84 ± 4.7	83 ± 4.2	83 ± 4.1	82 ± 3.3	82 ± 3.6	82 ± 3.3	80 ± 3.5	82 ± 3.5	81 ± 3.1	80 ± 2.8	80 ± 3.1	80 ± 2.4	81 ± 2.0	79 ± 2.5	79 ± 2.4	82 ± 2.2	82 ± 1.7	82 ± 2.0	84 ± 1.6	83 ± 1.7

*p < 0.05, **p < 0.01, ***p < 0.001, Sham compared with the TBI + vehicle

#p < 0.05, ##p < 0.01, ###p < 0.001, Sham compared with the TBI + AMN082

$p < 0.05, TBI + vehicle compared with the TBI + AMN082

### Treatment with AMN082 a TBI-induced motor impairment on the 3rd day after TBI

**Fig. 2:**
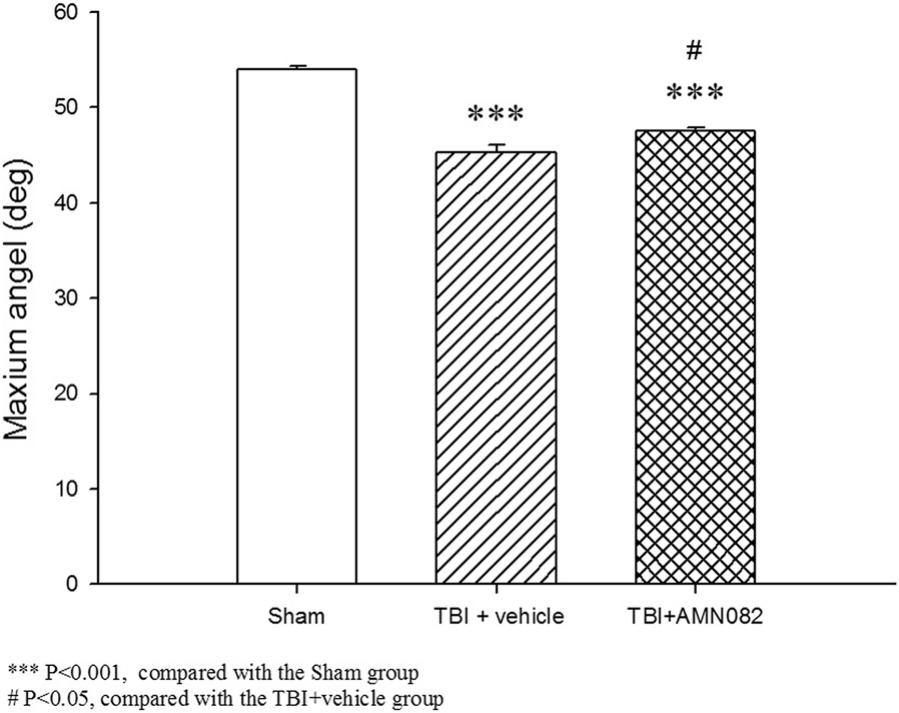
Effects of AMN082 on TBI-induced motor deficits on the 3rd day after TBI. ***p < 0.001, the sham group compared with the TBI group; ###p < 0.05, the sham group compared with the AMN082-treated TBI + group; $p < 0.005, the TBI group compared with the AMN082-treated TBI group; n = 6 in each group

The maximal grip angle of the TBI group was significantly lower than that of the sham controls on the 3^rd^ day after TBI (45.3° ± 0.72° versus 53.9 ± 0.37°, ***p < 0.001). TBI-induced motor impairment was significantly ameliorated by AMN082 treatment (the TBI group versus the AMN082 group, 45.3° ± 0.72° versus 47.5 ± 0.34, ^$^p < 0.05; n = 6 in each group; Fig. 2).

### Treatment with AMN082 significantly decreased the TBI-induced cerebral infarct volume on the 3rd day after TBI

**Fig. 3:**
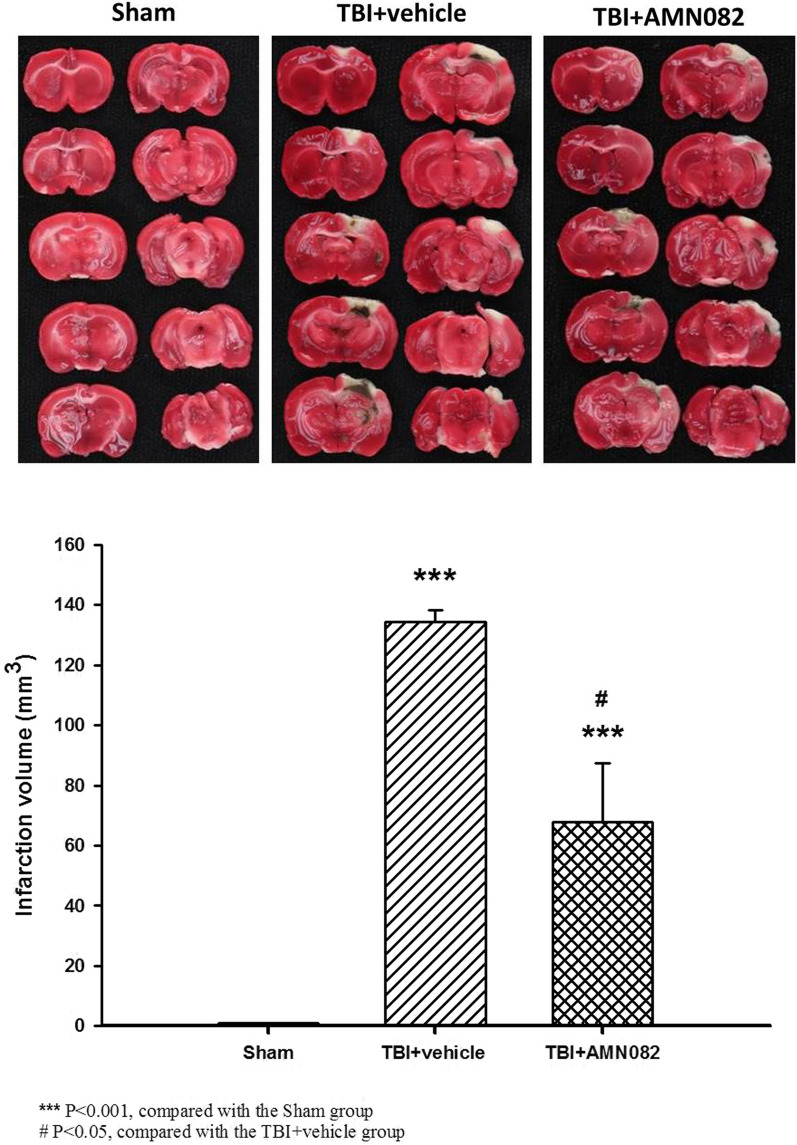
Effects of AMN082 on the TBI-induced infarct volumes in the pericontusional cortex on the 3rd day after TBI. ***p < 0.001, the sham group compared with the TBI group; #p < 0.05, the sham group compared with the AMN082-treated TBI + group; n = 6 in each group

Compared with the sham group, the TBI group showed a significant increase in infarct volume (134.4 ± 3.93 mm^3^ versus 0 ± 0 mm^3^, ^***^p < 0.001). However, compared with the TBI group, the AMN082-treated TBI group exhibited a significant reduction in the infarct volume (66.9 ± 19.62 mm^3^ versus 134.4 ± 3.93 mm^3^, ^#^p < 0.05; n = 6 in each group; Fig. 3).

### Treatment with AMN082 significantly attenuated neuronal apoptosis in the pericontusional cortex on the 3rd day after TBI

**Fig. 4:**
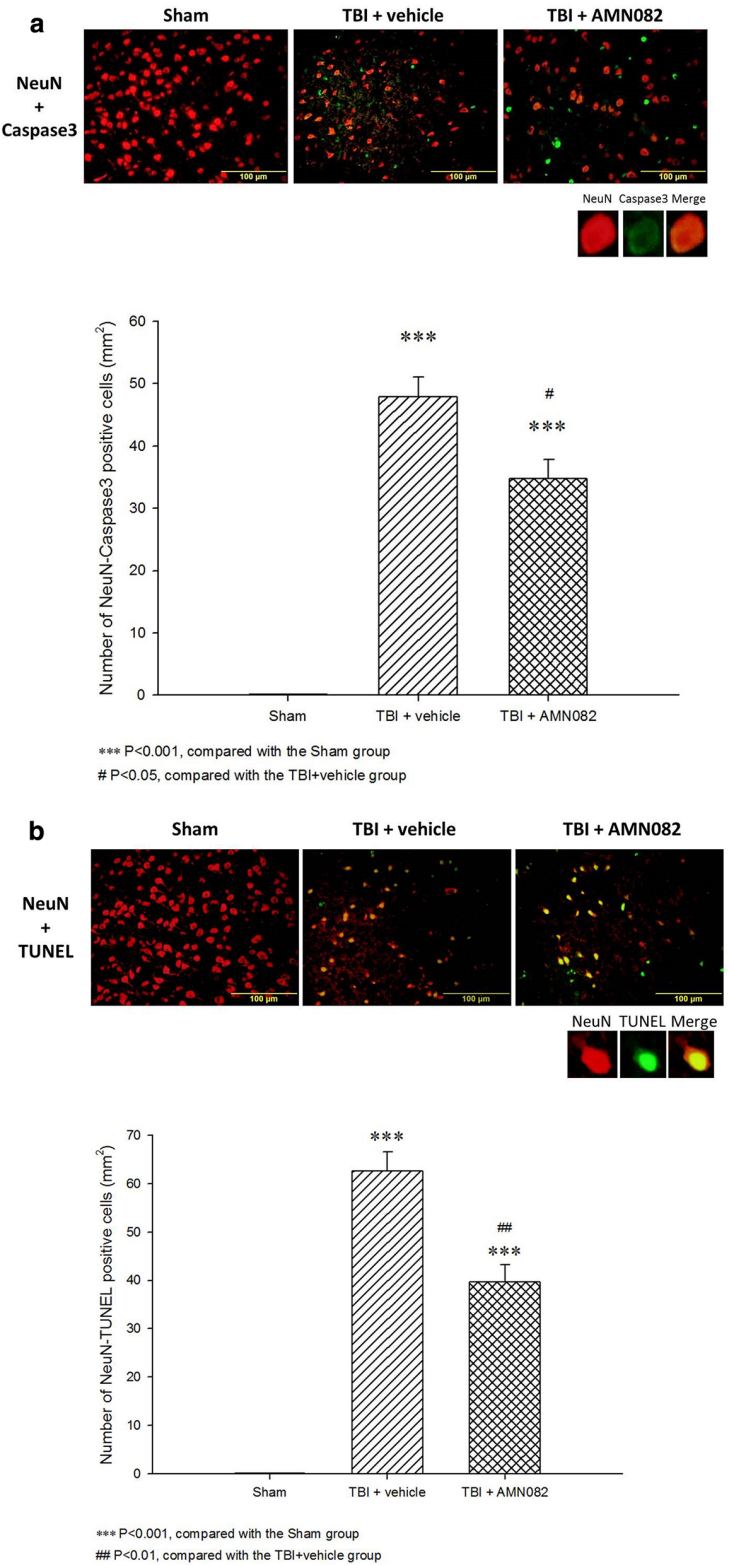
Effects of AMN082 on TBI-induced neuronal apoptosis in the pericontusional cortex on the 3rd day after TBI. Expression of the markers Neu-N and Caspase-3. ***p < 0.001 compared with the sham group; #p < 0.05 compared with the TBI group; n = 6 in each group (**a**). Expression of the marker Neu-N and TUNEL staining. ***p < 0.001 compared with the sham group; ##p < 0.01 compared with the TBI group; n = 6 in each group (**b**)

Caspase-3 and TUNEL staining revealed that the number of apoptotic neuronal cells (in the Neu-N and Caspase-3 staining and Neu-N and TUNEL staining assays) in the pericontusional cortex was significantly increased in the TBI group compared with the sham group on the 3^rd^ day after TBI (***p < 0.001). The number of positive apoptotic cells in rats subjected to TBI was significantly reduced after AMN082 treatment (^#^p < 0.05, ^##^p < 0.01; n = 6 in each group; Fig. 4a, b).

### Treatment with AMN082 significantly alleviated neuronal nitrosative stress in the pericontusional cortex on the 3rd day after TBI

**Fig. 5:**
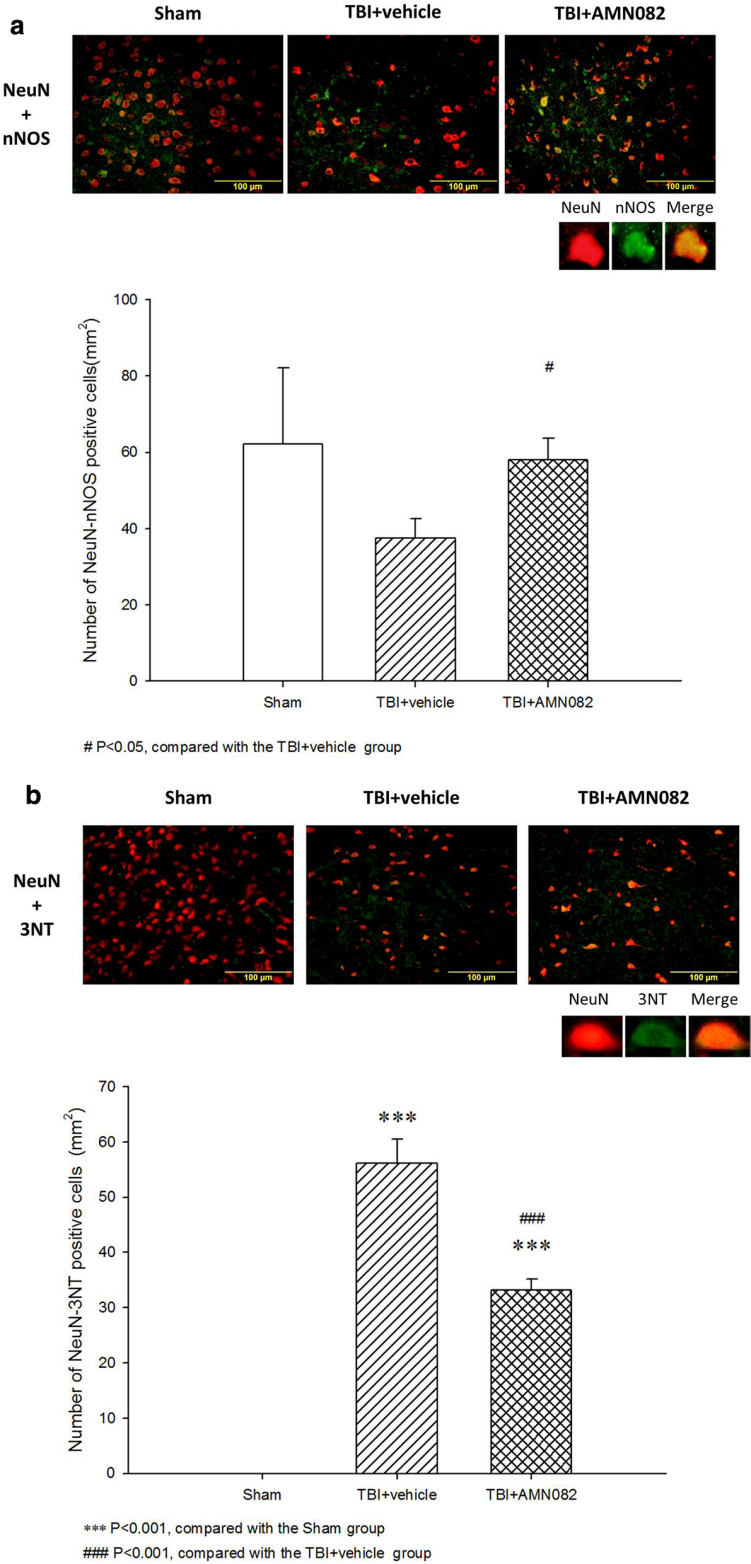
Effects of AMN082 on TBI-induced neuronal nitrosative stress in the pericontusional cortex on the 3rd day after TBI. Expression of the markers Neu-N and n-NOS. #p < 0.05 compared with the TBI group; n = 6 in each group (**a**). Expression of the markers Neu-N and 3-NT. ***p < 0.001 compared with the sham group; ##p < 0.01 compared with the TBI group; n = 6 in each group (**b**)

Compared with that in the sham controls (62.1 ± 19.87), the number of n-NOS-Neu-N-positive cells in the pericontusional cortex in TBI rats (37.6 ± 4.99; Fig. 5a) was nonsignificantly decreased. 3-NT and Neu-N staining revealed that the number of 3-NT-positive neuronal cells in the pericontusional cortex in vehicle-treated rats was significantly increased compared with that in the sham controls (56.0 ± 4.35 versus 0 ± 0; Fig. 5b). The TBI-induced decrease in the number of n-NOS-positive neurons and increase in the number of 3-NT-positive cells were significantly ameliorated by AMN082 therapy.

### Treatment with AMN082 significantly alleviated glial nitrosative stress in the pericontusional cortex on the 3rd day after TBI

**Fig. 6:**
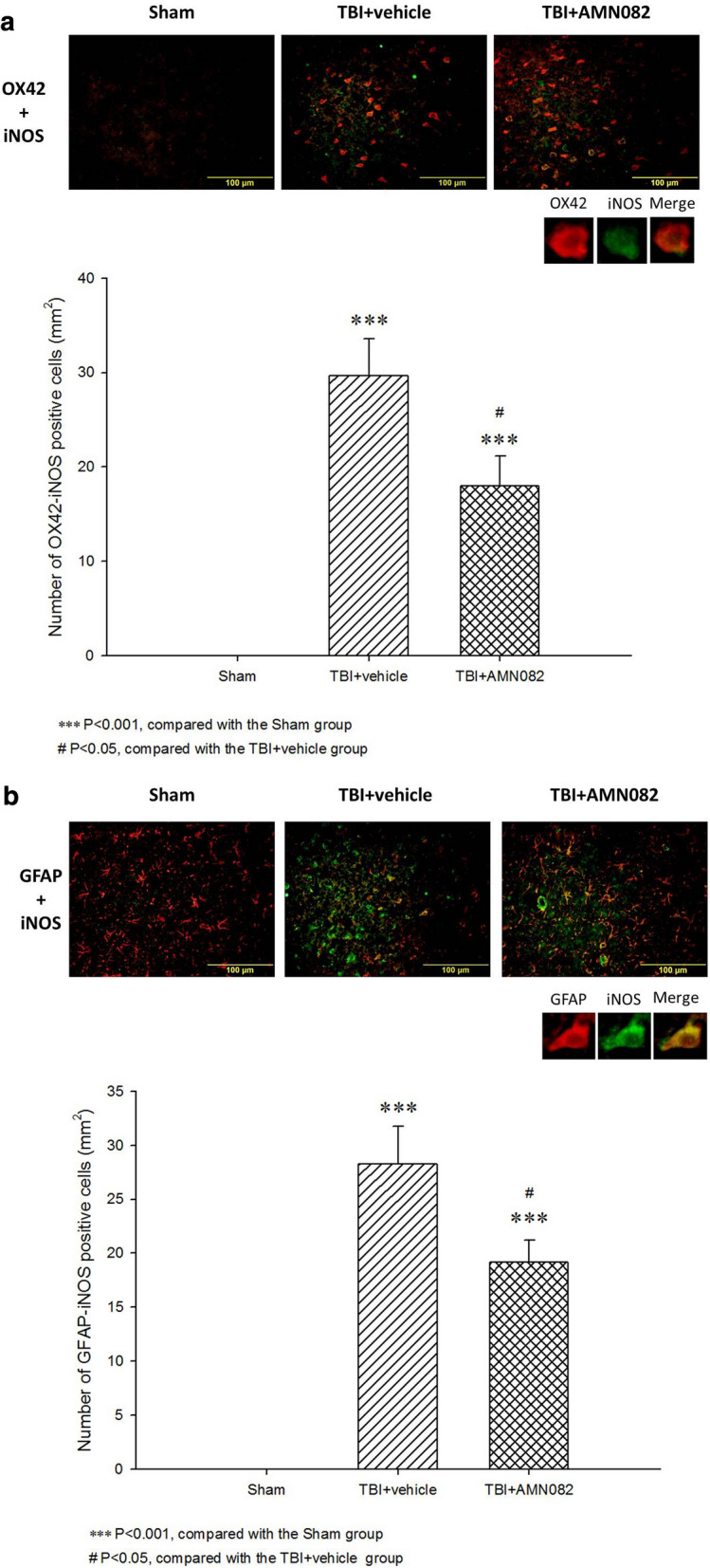
Effects of AMN082 on TBI-induced nitrosative stress in glia in the pericontusional cortex on the 3rd day after TBI. Expression of the markers OX-42-N and iNOS. ***p < 0.001 compared with the sham group, #p < 0.05 compared with the TBI group; n = 6 in each group (**a**). Expression of the markers GFAP and iNOS. ***p < 0.001 compared with the sham group; #p < 0.05 compared with the TBI group; n = 6 in each group (**b**)

TBI-induced iNOS expression in activated microglia (29.6 ± 3.92, ***p < 0.001; Fig. 6a) and astrocytes (28.2 ± 3.48, ***p < 0.001; Fig. 6b) was significantly lower in the AMN082-treated rats than in the control-treated TBI rats (17.9 ± 3.14 versus 19.1 ± 2.06, ^#^p < 0.05; n = 6 in each group).

### AMN082-treated rats had a higher NR2A/NR2B ratio in the pericontusional cortex than control-treated TBI rats

**Fig. 7:**
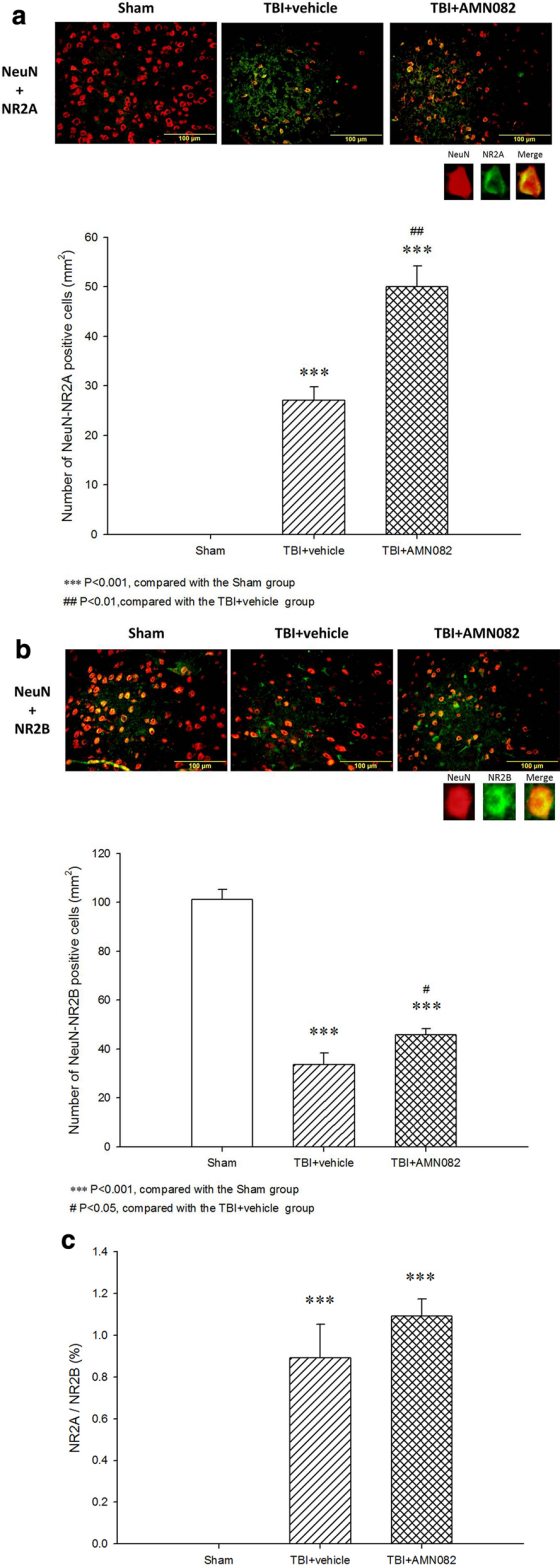
Effects of AMN082 on TBI-induced NR2A and NR2B expression in the pericontusional cortex on the 3rd day after TBI. Expression of the markers NeuN and NR2A. ***p < 0.001 compared with the sham group; ##p < 0.01 compared with the TBI group; n = 6 in each group (**a**). Expression of the markers NeuN and NR2B. ***p < 0.001 compared with the sham group; #p < 0.05 compared with the TBI group; n = 6 in each group (**b**). Compared with the vehicle-treated TBI group, the AMN082-treated TBI group had a higher NR2A/NR2B ratio (1.09 ± 0.08 versus 0.89 ± 0.16) (**c**)

Compared with that in the sham control group (0 ± 0), the number of NR2A- and Neu-N-positive cells in the pericontusional cortex in TBI rats (27.1 ± 2.74, ***p < 0.001; Fig. 7a) was significantly increased. The AMN082-treated group exhibited a significantly increased number of neuronal NR2A-positive cells after TBI (50.0 ± 4.18, ^##^p < 0.01; n = 6 in each group; Fig. 7a).

Compared with that in the TBI group, the number of NR2B- and Neu-N-positive cells (33.6 ± 4.76; n = 6; Fig. 7b) was significantly lower than that in the sham group (101.1 ± 4.03, ***p < 0.001; n = 6) and AMN082-treated group (45.7 ± 2.47, ^#^p < 0.05; n = 6).

We further measured the NR2A/NR2B ratio in the vehicle-treated TBI and AMN082-treated TBI groups, and we found that the AMN082-treated TBI group had a higher ratio than the vehicle-treated TBI group (1.09 ± 0.08 versus 0.89 ± 0.16; Fig. 7c).

## Discussion

### Summary of the current study

According to our review of the literature, this study is the first to demonstrate the effects of AMN082 on TBI. The results of this study add new information to the field of traumatology. We found that treatment with AMN082 improved neuropathology and functional outcomes after TBI. At the cellular level, AMN082 decreased iNOS and 3-NT expression, decreased neuronal apoptosis, and increased NR2A/2B glutamic receptor expression in astroglia and neuronal cells. Regarding functional outcomes, the AMN082-treated TBI group exhibited a very significant improvement in motor function. Therefore, we suggest that AMN082 may be a promising treatment agent for the treatment of TBI-induced nitrosative stress-associated neuronal apoptosis, which leads to functional impairment.

### Dosage of AMN082 and time course of AMN082 treatment

Several dosages of AMN082 and treatment durations are used in the neurobehavior field. AMN082 (1 mg/kg, I.P.) exerts significant antidepressant-like effects in the tail suspension test [29]. AMN082 (1 and 3 mg/kg, I.P.) decreases haloperidol (0.25 mg/kg)-induced parkinsonian-like effects [20]. AMN082 at doses of 2.5 and 5 mg/kg (I.P.) also decreases ethanol- and morphine withdrawal-induced anxiety-like behavior in the elevated plus-maze test [19]. AMN082 at a dose of 10 mg/kg (I.P.) suppresses the locomotor effect of both cocaine- and morphine-induced hyperactivity [21] When AMN082 is injected I.P. at a concentration of 10 ml/kg, activation of the mGlu7 receptor elicits antidepressant-like effects [30]. In the current study, we administered 10 mg/kg AMN082 for three consecutive days after TBI. These dose and time point of 3 days after TBI were selected because lateral fluid percussion causes motor dysfunction from 3 days to 1 year after TBI [31], and considerable evidence suggests that the brain edema volume peaks 2–3 days after TBI, which is usually when the ICP also reaches a maximum [32]. Our results support the idea that AMN082 at this dose may have therapeutic effects against TBI.

### Early effects of AMN082 on physiological parameters during the initial 120 min after TBI

In the current study, there was a trend for the AMN082 (10 mg/kg, I.P.)-treated group to have a lower ICP and higher CPP than the TBI group, specifically at approximately 0 min of the initial 120-min period after TBI. We speculate that these early beneficial effects of AMN082 on ICP and CPP may lead to attenuation of secondary injury and improvements in outcomes on the 3^rd^ day after TBI. Sukoff Rizzo et al. demonstrated that following a single injection of 10 mg/kg AMN082 (I.P.), the drug crossed the blood–brain barrier and reached peak concentrations in the brain 30 min posttreatment [10]. However, we did not investigate the relationship between the AMN082 concentration in the brain and changes in physiological parameters after TBI. Therefore, this relationship warrants further investigation.

### Possible mechanisms of action of AMN082 against TBI-relate neuropathological changes and functional outcomes

Inducible NOS produces large amounts of nitric oxide (NO) and then causes the generation of 3-NT, leading to cell apoptosis [6, 33]. The occurrence of such nitrosative stress-associated neuronal apoptosis in in vivo and in vitro TBI models is consistent with peroxynitrite-mediated inhibition of the effects of caspase [33]; brain cooling [6] or memantine [5] can attenuate brain nitrosative damage, decrease the infarct volume in the cortex and improve functional outcomes after TBI, and coculture with 3NT induces motor neuron apoptosis [34]. In the current study, we found that the expression of iNOS was increased in microglia and astrocytes, neuronal apoptosis was increased in the TBI group, and these parameters were attenuated in the AMN082-treated TBI group (Table 2). Therefore, we speculate that the neuroprotective effects of AMN082 may be related to its attenuation of the nitrosative stress-associated neuronal apoptotic pathway, which leads to functional impairment after TBI.

The glutamate receptor subunit NR2A is involved in neuroprotection, and NR2B triggers destructive pathways. Both NR2A and NR2B are mainly located in neurons [4]. To date, the expression of NR2A and NR2B following AMN082 treatment after TBI has not been investigated. In the current study, we found that the expression of both NR2A and NR2B was significantly increased in the AMN082-treated TBI group and that the AMN082-treated TBI group had a higher NR2A/NR2B ratio than the vehicle-treated TBI group (Table 2). Therefore, we speculate that AMN082 may affect NR2A and NR2B expression, although AMN082 is a highly selective mGlu7 receptor agonist. However, the detailed mechanisms need to be investigated in the future.

Neuronal nitric oxide synthase (nNOS) is the predominant source of NO in neurons and has dual biological activities [35]. Its activity is related to NMDA receptor-mediated excitotoxicity [36]. In the current study (Table 2), we found that AMN082 increased nNOS expression while reducing both neuronal 3-NT expression and apoptosis and increasing the NR2A/NR2B ratio, which may be an indicator of neuroprotection [5]. Under such conditions, nNOS may play a role in neuroprotection, and nNOS may be activated by AMN082 administration.

Therefore, we infer that there are several possible mechanisms of action that may be involved. First, AMN082, a highly selective mGlu7 receptor agonist, inhibits presynaptic glutamate release from rat cerebrocortical nerve terminals [5]. Therefore, it appears to inhibit nitrosative stress development and exert neuroprotective effects by reducing glutamate release into the postsynaptic space or extrasynaptic space. Second, AMN082, through postsynaptic mGlu7 receptors, can modulate NMDA receptor activity [17, 37] or directly bind to NMDA receptors such as NR2A and NR2B-containing receptors, resulting in neuroprotection [5, 10]. Third, AMN082 binds to the mGlu7 receptor in astrocytes and microglia [23] and attenuates TBI-induced iNOS expression in activated microglia and astroglia in the pericontusional cortex. These possible mechanisms may result in reductions in neuronal 3NT expression and apoptosis and ultimately improve functional outcomes after TBI. Although there are various possibilities, the results show that AMN082 is an effective and practical agent for TBI treatment, particularly in that it exerts neuroprotective effects by attenuating nitrosative stress-associated apoptosis in the brain, which is a serious form of secondary injury after TBI.

### Limitations of the current study

Several drawbacks of the current study should be mentioned. First, we only investigated the NMDA receptor subunits NR2A and NR2B but did not assess the expression of kainite or α-amino–3-hydroxy-5 methyl-4-isoxazole propionic acid (AMPA) receptors, which may influence nitrosative stress and apoptosis after TBI. Second, we only provided proof of associations between the parameters at a specific time point, i.e., 3 days, after TBI. The long-term effect of AMN082 on TBI needs to be clarified in the future. Third, we only assessed the effects of AMN082 in male rats; it should be determined whether these results can be generalized to female rats or other species and whether they are applicable to clinical practice in the future. Fourth, we only used a single functional test, the inclined plane test, to evaluate the motor functions of rats after neural damage. However, our results showed a small absolute difference in angle between the TBI + vehicle and TBI + AMN082 groups. Whether these results indicate a clinically meaningful difference in humans needs further confirmation. Additionally, multiple tests of motor function should be used in the future. Finally, this study included 3 groups: the sham-operated group, vehicle-treated TBI group and AMN082-treated TBI group. Because AMN082 has not been applied in TBI, these novel data regarding the effects of AMN082 on neurons and glia as well as TBI-induced nitrosative stress-associated neuronal apoptosis and neuronal NR2A/2B expression may be useful for critical care physicians in the clinic. However, we could consider adding another group subjected to TBI and treated with an AMN082 antagonist group, such as 6-(4-methoxyphenyl)-5-methyl-3-pyridinyl-4-isoxazolo [4,5-c] pyridin-4(5H)-one (MMPIP) [38], which is a selective mGluR7 antagonist; this addition would make the results stronger and more convincing. Therefore, many more studies are required in the future.

## Conclusion

Our results demonstrate that intraperitoneal administration of AMN082 for 3 days after TBI may ameliorate TBI insults by affecting glutamate receptor-related nitrosative stress and NR2A/2B expression and decreasing neuronal apoptosis in the injured cortex. These mechanisms might explain the observed functional recovery. We also speculate that AMN082 may be a promising agent for the treatment of TBI in the acute stage.

## Data Availability

The datasets used and/or analyzed during the current study are available from the corresponding author upon reasonable request.

## Abbreviations

TBI: Traumatic brain injury
SD: Sprague–Dawley
TTC: Triphenyltetrazolium chloride
TUNEL: Terminal deoxynucleotidyl transferase-mediated dUTP-biotin nick end labeling
GFAP: Glial fibrillary acidic protein
DAPA: 4′, 6-Diamidino-2-phenylindole
HR: Heart rate
MAP: Mean arterial pressure
ICP: Intracranial pressure
CPP: Cerebral perfusion pressure
NR2A: *N*-Methyl-d-aspartate receptor 2A
NR2B: *N*-Methyl-d-aspartate receptor 2B
mGlu: Metabotropic receptor
iNOS: Inducible nitric oxide synthase
